# Ultrasound findings in a case of Eales’ disease and ocular trauma with anterior chamber cholesterolosis

**DOI:** 10.1186/s12886-020-01660-1

**Published:** 2020-10-06

**Authors:** Peng Lu, Jingjing Huang

**Affiliations:** State Key Laboratory of Ophthalmology, Department of Glaucoma, Zhongshan Ophthalmic Center, Sun Yat-sen University, 7 Jinsui Road, Guangzhou, 510623 China

**Keywords:** Cholesterolosis, Cholesterol crystal, Eales’ disease, Ocular trauma, Ultrasound

## Abstract

**Background:**

Anterior chamber cholesterolosis is a rare phenomenon which occurs mostly in chronically blind eyes. We present the anterior and posterior ultrasound findings in a case of anterior chamber cholesterolosis secondary to Eales’ disease and ocular trauma, which may contribute to the understanding of the potential mechanism of this phenomenon.

**Case presentation:**

A 48-year-old man presented with “sparking” right eye, which appeared soon after the ocular trauma. Both eyes were confirmed Eales’ disease in our center 8 years ago, and right eye remained no light perception since then. Intraocular pressure of right eye measured by Goldmann applanation tonometry was 1 mmHg. Slitlamp photograph revealed multiple polychromatic large crystals in anterior chamber. Ultrasound biomicroscopy showed that anterior chamber was filled with extensive large granular substances. Dense dotted hyperechoic foci and retinal detachment was found in B-scan ultrasound examination. The right eye was diagnosed as anterior chamber cholesterolosis secondary to Eales’ disease and ocular trauma. The patient was asymptomatic, and therefore was advised to have regular follow-up.

**Conclusion:**

The findings of above imaging examinations, as well as complaint of “sparkling” eye appeared soon after ocular trauma elucidate that anterior chamber cholesterol crystals were from vitreous cavity. Any factors facilitating the communication of anterior chamber and vitreous body may lead to the occurrence of this rare phenomenon in predisposing eyes. The anterior and posterior ultrasound findings may give a clue on the potential mechanism.

## Background

Anterior chamber cholesterolosis, with polychromatic shining crystals presenting in anterior chamber, is an interesting and uncommon condition which can occur in eyes suffering from vitreous hemorrhage, hyphema, long-standing retinal detachment, chronic intraocular inflammation, and Coats disease [1–5]. Anterior chamber cholesterolosis secondary to Eales’ disease is extremely rare [6]. Limited imaging information confined our understanding of this phenomenon [6].

We report a case of anterior chamber cholesterolosis in a 48-year-old patient suffered from Eales’ diseases and ocular trauma, showing the anterior and posterior ultrasound findings. Notably, this is the first description of the characteristics of ultrasound biomicroscopy (UBM) findings of anterior chamber cholesterolosis. The case report may enable us to understand the potential mechanism of this phenomenon.

## Case presentation

A 48-year-old man presented with “sparkling” right eye for 1 year, without redness and pain. His right eye was punched by a hoe 1 year ago and remained untreated. Soon after that, the affected eye showed “sparking” appearance.

According to his medical record documented 8 years ago, his right eye remained no light perception since then, and intraocular pressure was high (57 mmHg) due to neovascular glaucoma. Dilated fundus examination and B-scan ultrasound indicated vitreous hemorrhage, while retinal detachment was not found. Fundus fluorescein angiography (FFA) revealed engorged tortuous veins, diffused vascular occlusion area and lots of microangiomas on the edge of inferior-nasal occlusion area in the right eye, and slightly engorged tortuous veins with increased permeability in the left eye. The patient reported no systemic diseases such as hypertension, diabetes mellitus, sickle cell disease. The diagnosis of Coat’s disease, retinal vein occlusion, diabetic retinopathy and sickle cell disease were excluded according to the fundus examination, FFA results and medical history [7, 8]. A final diagnosis of Eales’ disease on both eyes and neovascular glaucoma on right eye was made. Right eye was treated with photocoagulation, and systemic and topical anti-glaucoma therapy. The patient did not come back to follow up until 8 years later.

**Fig. 1:**
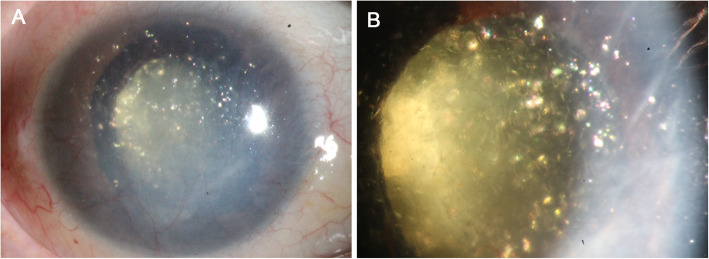
Slitlamp photograph of right eye showed multiple polychromatic shining crystals drifting in anterior chamber

**Fig. 2:**
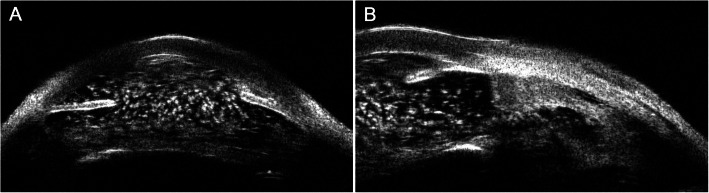
Ultrasound biomicroscopy of right eye revealed (**a**) anterior chamber was filled with granular substances and (**b**) detachment of ciliary body

**Fig. 3:**
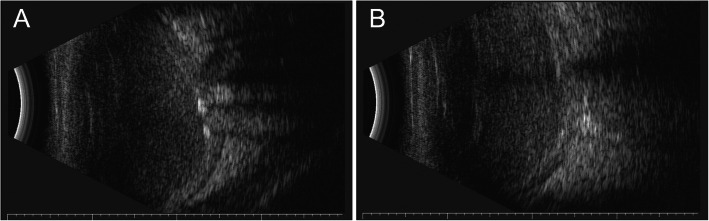
B-scan ultrasound of right eye revealed dense dotted hyperechoic foci in vitreous cavity and retinal detachment

At the current visit, right eye was no light perception and intraocular pressure measured by Goldmann applanation tonometry was 1 mmHg. On examination, multiple polychromatic glistening large crystals were found to float in the slightly straw yellow-colored fluid (Fig. 1). As shown in UBM examination, the central anterior chamber depth is 3.83 mm, which is much larger when compared with the contralateral eye (2.75 mm), anterior chamber angle is closed in all positions, iris are markedly thin and stiff, masses of large granular substances exist in the anterior chamber (Fig. 2a), the ciliary body and anterior choroid are detached (Fig. 2b). B-scan ultrasound examination revealed dense dotted hyperechoic foci and retinal detachment (Fig. 3). The patient was diagnosed with anterior chamber cholesterolosis secondary to Eales’ disease and ocular trauma in his right eye based on the above findings. However, the right eye was no light perception and asymptomatic, the patient was advised to have regular follow-up.

## Discussion and conclusions

Anterior chamber cholesterolosis is a secondary phenomenon which can rarely occur in blind eyes suffering from severe ocular trauma or degenerative ocular diseases, for example, Coats’ disease [1, 2]. Unlike Coats’ disease, Eales’ disease is an idiopathic retinal periphlebitis characterized by three basic pathological changes: inflammation, ischemia, and neovascularization [9]. Ocular ischemia caused by Eales’ disease may account for the development of neovascular glaucoma in this case. Recurrent vitreous hemorrhage from retinal neovascularization is a hallmark of Eales’ disease and may lead to tractional or combined retinal detachment [9]. The anterior chamber cholesterolosis secondary to Eales’ disease is even rarer.

Cholesterol crystals are considered to originate from lipid-rich cell membranes of erythrocytes during the breakdown of vitreous hemorrhage [1]. Long-standing retinal detachment without intraocular hemorrhage may also be the cause of cholesterol crystals formation in some eyes, because subretinal fluid is also rich in lipid [1]. In our case, vitreous hemorrhage caused by Eales’ disease and the consequent retinal detachment might together contribute to the formation of cholesterol crystals.

A definite diagnosis of cholesterol crystals can be made through histopathologic examination. Cholesterol crystals will demonstrate birefringence with polarized light and positive staining with lipid stains such as Oil-Red-O test [1, 10]. However, clinical diagnosis of cholesterol crystals is easy to make because of its typical characteristics and limited differential diagnosis including calcium oxalate crystals, proteinaceous crystals and aqueous cells. Cholesterol crystals are multicolored and much larger than the above three substances [1]. In addition, proteinaceous crystals and aqueous cells tend to occur in phacolysis, but cholesterol crystals are usually found in chronically blind eyes [1]. Anterior chamber cholesterol crystals of our case revealed by slitlamp photograph were in line with previous studies [1, 10]. Notably, masses of large granular substances as shown in UBM images of the current case might further support the diagnosis of anterior chamber cholesterolosis.

A hypothesis for the pathogenesis of anterior chamber cholesterolosis is that cholesterol crystals have already formed in the vitreous cavity, and ocular trauma, which may lead to the dysfunction of suspensory ligament and subsequent lens (sub) luxation, will allow cholesterol crystals entering anterior chamber from vitreous cavity [1–3]. Asymmetry of the central anterior chamber depth is an important clinical feature of the lens subluxation [11]. Our patient suffered from an ocular trauma, which could be a trigger factor, and showed a significant difference in the depth of central anterior chamber (3.83 mm of the affected eye vs. 2.75 mm of the unaffected eye), and masses of large granular substances in the anterior chamber without any lens echo in the UBM images. The asymmetry of the central anterior chamber depth of the current case might be caused by lens subluxation or the absorption of lens. The findings of B-scan ultrasound showed extensive hyperechoic foci in vitreous body. Additionally, glistening substances were found in anterior chamber soon after the ocular trauma. Taking all together, our case may evidence this hypothesis.

Secondary glaucoma is a common complication of anterior chamber cholesterolosis and occurred in most previously reported cases [5, 12]. However, the intraocular pressure of the current case was low, probably because of the detachment and degeneration of the ciliary body.

In conclusion, cholesterol crystals may have already existed in the vitreous body of predisposing eyes. Any factors that can facilitate the communication of the anterior chamber and vitreous body may lead to the occurrence of anterior chamber cholesterolosis. The non-invasive UBM examination can demonstrate the characteristics of anterior chamber cholesterolosis and help us understand this rare phenomenon.

## Data Availability

Data sharing is not applicable to this article as no datasets were generated or analysed during the current study.

## Abbreviations

UBM: Ultrasound biomicroscopy
FFA: Fundus fluorescein angiography
